# Rehabilitation using single stage implants

**DOI:** 10.4103/0972-124X.51893

**Published:** 2009

**Authors:** Jumshad B. Mohamed, Sabitha Sudarsan, K. V. Arun, B. Shivakumar

**Affiliations:** 1 *Postgraduate Student, Department of Periodontics and Implant Dentistry, Ragas Dental College and Hospital, Uthandi, Chennai, India*; 2 *Professor and Head, Department of Periodontics and Implant Dentistry, Ragas Dental College and Hospital, Uthandi, Chennai, India*; 3 *Professor, Department of Periodontics and Implant Dentistry, Ragas Dental College and Hospital, Uthandi, Chennai, India*; 4 *Reader, Department of Periodontics and Implant Dentistry, Ragas Dental College and Hospital, Uthandi, Chennai, India*

**Keywords:** Compromised ridge, implant, single stage

## Abstract

Implant related prosthesis has become an integral part of rehabilitation of edentulous areas. Single stage implant placement has become popular because of its ease of use and fairly predictable results. In this paper, we present a series of cases of single stage implants being used to rehabilitate different clinical situations. All the implants placed have been successfully restored and followed up for up to one year.

## INTRODUCTION

Osseo-integrated implants provide predictable restorative support for crowns, restoration, prosthesis abutment, and removable dentures. Their widespread use in recent years has resulted in an increasing percentage of the adult population with implant supported prosthesis. With the two-stage devices, the prosthetic loading procedures are usually instituted four to six months after initial insertion of the implant. Although time tested and predictable, two-stage implants suffer from limitations of: protracted time of procedure b) inevitable loss of tissue that occurs during second stage procedure. To overcome these difficulties, single stage implants were introduced with the advantage of increased patient acceptance and shortened procedure time.[1,2] Rapid loading protocol for aesthetic single-tooth replacement can increase patient acceptance by limiting multi-step process of dental implant therapy.[3,4] Mauro Donati *et al*.[5] reported that immediate functional loading of implants placed with a conventional installation technique and with sufficient primary stability may be considered a valid treatment alternative in a single-tooth replacement. Fritz *et a1*.[6] evaluated the success of implants in regenerated bone from a histological perspective and reported no significant difference between native bone and regenerated bone sites after loading with a fixed prosthesis for one year. In a meta-analysis of 13 prospective trials done, Effie Ioannidou *et al*.[7] concluded that early implant loading was not associated with worse outcomes compared to conventional loading. This paper presents a series of reports of rehabilitation using single stage implants.

## CASE REPORTS

### Case I:

**Figure 1:**
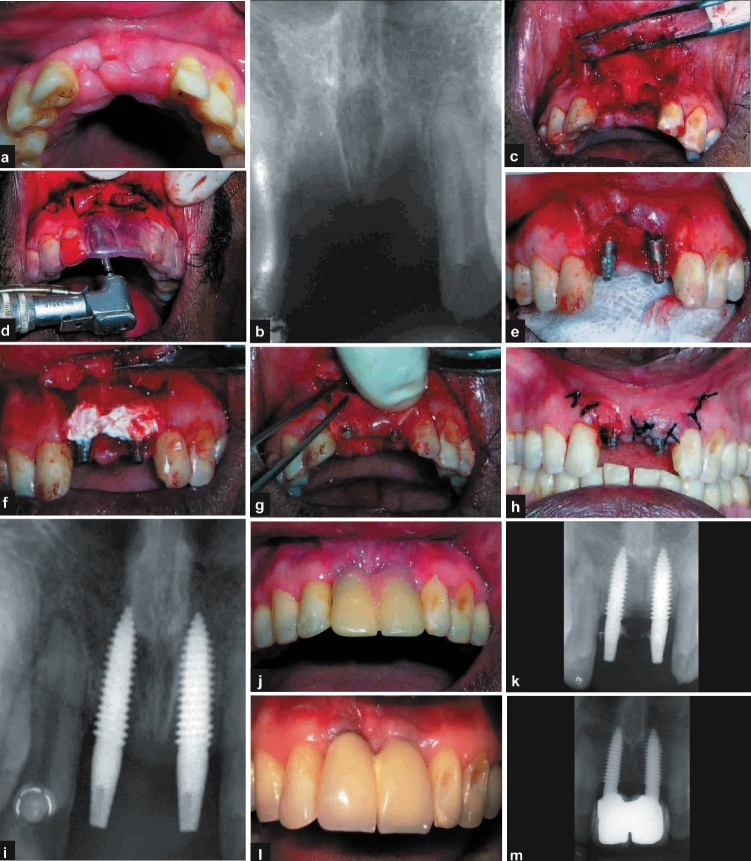
a, b) Pre-operative photograph and radiograph; c- i) operative photographs and radiograph; j-m) post-operative photographs and radiographs

A 41-year-old male patient presented with a history of trauma and subsequent avulsion of tooth number 11 and crown fracture at the neck of tooth number 21, previously extracted two weeks prior, at another dental facility. The patient requested an immediate fixed solution to the lost anterior teeth from our department. Clinical and radiological evaluation revealed adequate apico-occlusal alveolar bone, but extensive loss of the labial plate [Figure 1a and b]. The soft tissue dimensions were, however, adequate for functional and aesthetic acceptability. It was decided to perform a single stage immediate loading implant placement with simultaneous augmentation of the lost facial plate using calcium phosphate bone substitute* and collagen barrier#.

After induction of local anaesthesia, full thickness flap was reflected in relation to number 11 and number 21. The sockets were debrided with curettes and two single piece implants$ (3.7 × 13 mm) were placed into the sockets. After checking for primary stability, which was achieved by wrenching the implant into the bone beyond the apex of the socket, calcium phosphate cement* was mixed in sterile saline and injected into the spaces between the implant and the socket walls. Collagen membrane# was trimmed to the appropriate size and shaped to cover the graft material. The flap was approximated and sutured with interrupted 3-0 Mersilk [Figure 1c–i]. The patient was instructed to discontinue tooth brushing and avoid trauma or pressure at the surgical site. A 0.2% chlorhexidine mouth rinse was prescribed two/three times daily for two-three weeks following surgery, and antibiotics and anti-inflammatory drugs were prescribed for five days. The sutures were removed after 10 days and the patient received temporary acrylic crowns on the same day. The patient was recalled for prophylaxis after two and four weeks and every three months. The clinical and radiographic appearances at six months and after one year show good aesthetic result and acceptable osseo-integration of the implants [Figure 1j–m].

#### Discussion

The presence of adequate palatal bone and the mesial and distal labial plates meant that we could achieve not only good primary stability following placement of implant, but also that the anatomical characteristics of defect would favour regeneration following placement of bone substitutes.[8] Existing literature suggests that long term prognosis of implant-related prosthesis would be directly affected by the primary stability achieved during placement.[5] Simultaneous bone augmentation procedure may be attempted only when native bone is sound enough for primary stability to be obtained.[9,10] If such were not the case, it would be prudent to augment the ridge conventionally using GBR procedure and subsequently place implant after four to six months.

### Case II:

**Figure 2:**
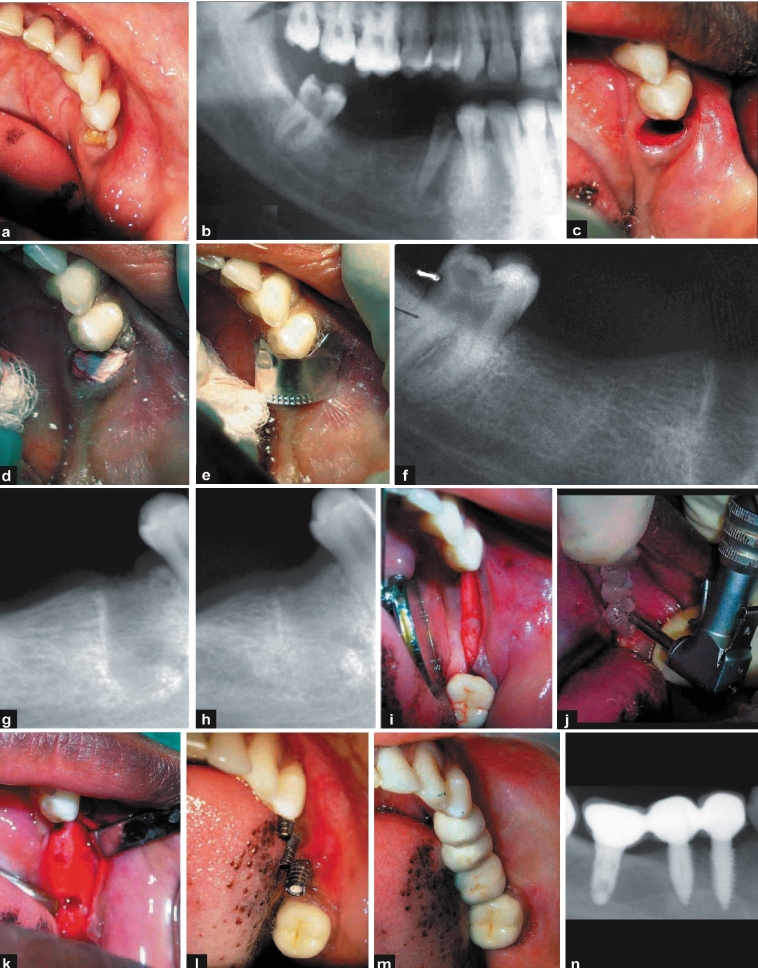
a, b) Pre-operative photograph and radiograph; c-f) operative photographs and radiograph during socket preservation; g, h) 1-month and three-month radiograph post socket preservation; i-k) implant placement photographs; l-n) post-operative photographs and radiograph

A 25-year-old female patient presented with a root stump in relation to 44 and missing tooth number 45 and 46. Various treatment options were given to the patient following which she decided to undergo implant supported prosthesis. Clinical and radiological evaluation of tooth number 44 revealed infected root stump with evidence of periapical pathology [Figure 2a and b]. The keratinised mucosa around number 44 was inadequate and deemed incompatible with long term prosthetic health. It was decided to initially debride the socket and augment the bone prior to implant placement in all three missing teeth. Surgical technique involved a traumatic extraction of root stump in relation to number 44 with periotomes. The socket was thoroughly debrided with curettes and excavators. Calcium phosphate bone substitute was injected into the socket up to the brim and no sutures were placed. A foil was trimmed to size and shape and placed over it and periodontal dressing was placed over it [Figure 2c–f]. Post-operative instructions were similar to the one described in case I. The healing occurred by creeping of the keratinised mucosa along the open wound margins and was closed at 14 days' time when the dressing and foil were removed.

After 10 weeks, clinical evaluation showed adequate keratinised mucosa and radiograph revealed good bone formation [Figure 2g and h]. Three months after socket grafting, the site relation to numbers 44 and 45 received single piece implant$ (3.7 × 10 mm) and number 46 received a two-piece implant (3.75 × 8 mm). The bone appeared quite similar to the adjacent bone during the osteotomy as bone adjacent to the site were also prepared to receive implants at the same time [Figure 2i–k]. The clinical appearance after six months reveals good aesthetics and adequate amount of keratinised mucosa, and radiographs revealed good osseo-integration of the implant [Figure 2l–n].

#### Discussion

The calcium phosphate bone substitute* used in this procedure obviates the necessity for primary closure when used in socket preservation cases.[8] The Hydrase (wetting agent) used to mix with calcium phosphate cement supports epithelial proliferation and subsequent keratinisation. Hence, using this procedure, we managed to preserve what little keratinised gingiva was left and also obtain keratinised mucosa in the crestal area. The presence of persistent periapical pathology may lead to retrograde periimplantitis[11,12] and is thought to be an important cause of failure in immediate placement implants. Pathology observed in the radiograph and its proximity to adjacent edentulous sites was the reason for delayed placement of the implant in this case. This way, we achieved good soft and hard tissue dimensions at the site of implant placement and also considerably reduced the potential for future retrograde periimplantitis.

### Case III:

**Figure 3:**
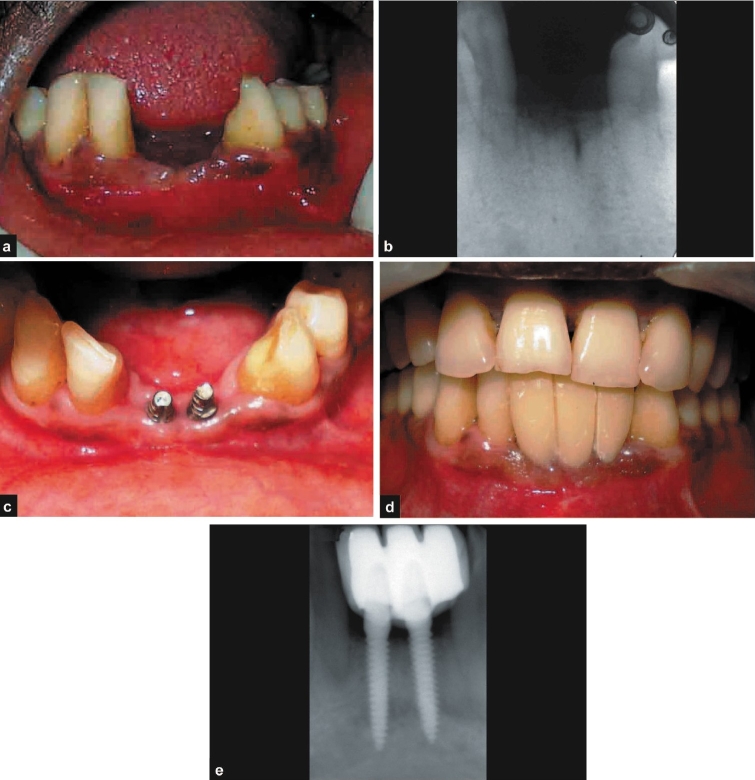
a, b) Pre-operative photograph and radiograph; c-e) post-operative photographs and radiograph

A 42-year-old male patient presented with a history of exfoliation of tooth number 31 and 41 and desirous of immediate replacement. After careful clinical and radiological evaluation, the patient was diagnosed with severe chronic periodontitis. Surgical periodontal therapy was performed and the patient placed on maintenance regime. After satisfactory maintenance of periodontal health was achieved and good oral hygiene practices were instituted by the patient, the patient was taken up for implant placement in relation to numbers 31 and 41. On examination of the edentulous site, there was a vertical and horizontal deficient ridge (Seibert's Class III) for which the patient was offered the option of initial augmentation followed by subsequent implant placement [Figure 3a and b]. The patient decided not to undergo the graft procedure and it was decided to arrive at a compromise by placing a smaller diameter single stage implant$ (2.8 × 13 mm). Postoperative protocols were similar to the previous case. The clinical and radiographic appearances at six months and one year show good aesthetic result and acceptable osseo-integration of the implants [Figure 3c–e].

#### Discussion

Strategic extraction[13] has become an important part of treatment planning for both periodontal and prosthetic purposes. The patient described in this case would have benefited from an earlier strategic extraction whereby adequate alveolar bone both in horizontal and vertical dimension would have been present to make room for a more aesthetic result.[14,15] It is also important to realize that the patient's wishes are paramount during the treatment planning stages for any procedure, especially for the more expensive procedures such as implant supported prosthesis.

### Case IV:

**Figure 4:**
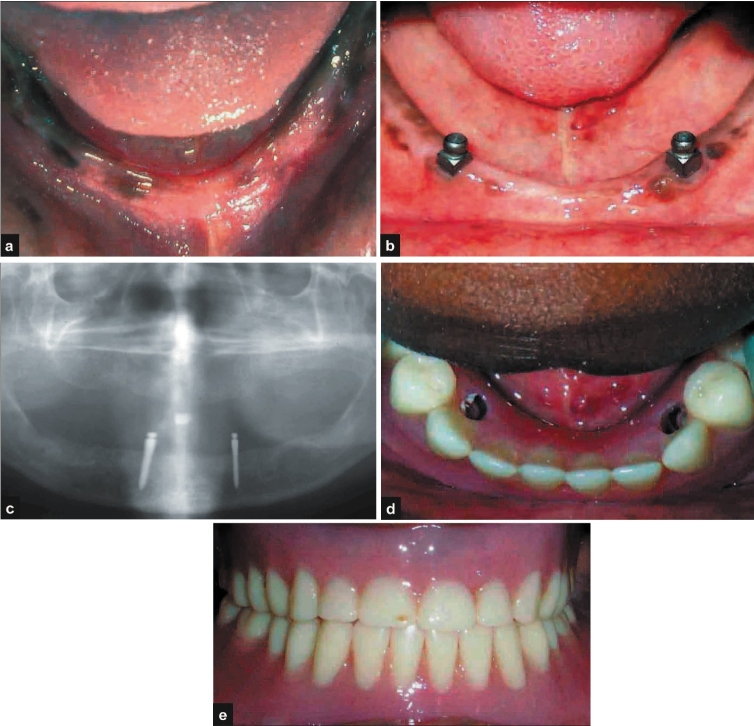
a) Pre-operative photograph; b-e) post-operative photographs and radiograph

A 25-year-old female patient presented with a history of complete loss of teeth and wearing complete dentures since six months. The patient requested a fixed option for the lower denture. Clinical and radiological evaluation was performed [Figure 4a]. It was decided to perform a single stage over denture implant placement. After induction of local anaesthesia, full thickness flap was reflected in relation to numbers 33 and 43. Two single piece over-denture implants$$ (2.4 × 13 mm) were placed. After checking for primary stability, the flap was approximated and sutured with interrupted 3-0 Mersilk. The patient was instructed to discontinue wearing the lower denture and avoid trauma or pressure at the surgical site. Post-operative instructions were similar to the ones described in the first case. The sutures were removed after 10 days and the patient's existing lower denture was modified to receive the female part of the ball and socket over-denture prosthesis. The patient was recalled for review after two and four weeks and every three months. The clinical and radiographic appearances at six months and at one year show good aesthetic result and acceptable osseo-integration of the implants [Figure 4b–e].

#### Discussion

The economic reality of implant-related prosthesis means that we may not be able to offer the required number of implants for perfect prosthetic rehabilitation especially if expensive bone regeneration procedures are required initially. In such instances, an implant supported over-denture may be an acceptable solution[16] providing greater patient relief in terms of denture support.[17]

### Case V:

**Figure 5:**
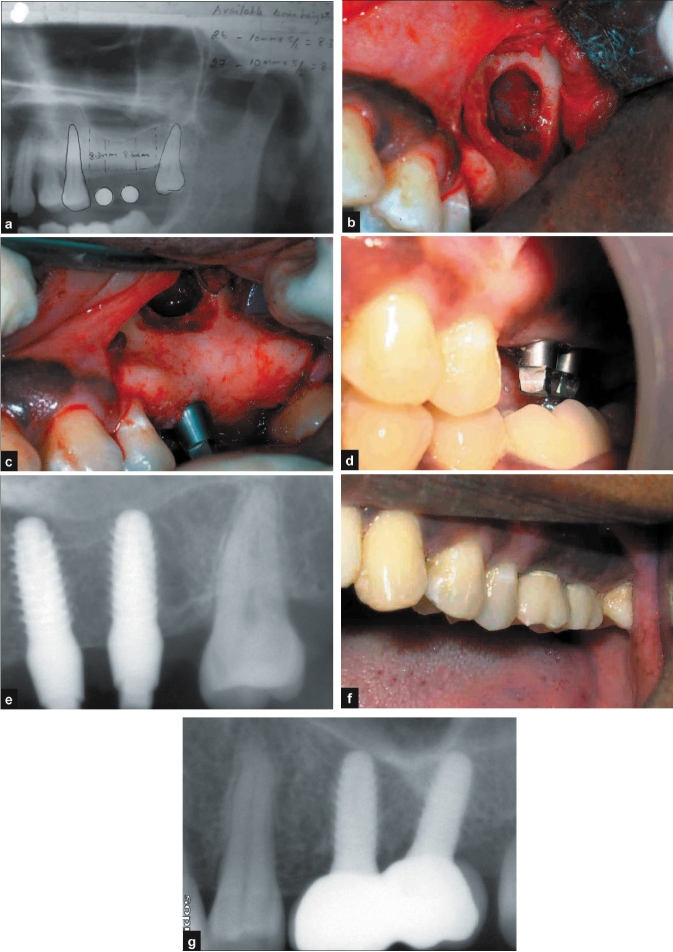
a) Pre-operative radiograph; b-e) operative photographs and radiograph; f, g) post-operative photograph and radiograph

A 35-year-old female patient presented with a history of loss of tooth number 26 and 27 due to periapical infection. The patient presented to our department requesting for a fixed solution for her lost teeth without disturbing the adjacent teeth. Clinical and radiological evaluation [Figure 5a] revealed available native bone height of 7-8 mm at both the sites. It was decided to perform a single stage implant placement with sinus lift (lateral approach). After induction of local anaesthesia, full thickness flap was reflected in relation to numbers 26 and 27. Two single piece implants$ (4.5 × 10 mm) were placed [Figure 5b–e]. After checking for primary stability, the flap was approximated and sutured with interrupted 3-0 Mersilk.

Post-operative protocols were similar to the one described in Case I. The sutures were removed after 10 days and the patient received temporary acrylic crowns on the same day. The patient was recalled after two and four weeks and every three months. The clinical and radiographic appearances at six months and one year show good aesthetic result and acceptable osseo-integration of the implants [Figure 5f and g].

#### Discussion

Implant placement in the posterior maxilla is often compromised by lack of adequate bone.[18] Sinus augmentation procedures are a viable option in such instances. Existing literature suggests that sinus augmentation with simultaneous implant placement may be successful provided the available bone is more than 3-5 mm.[19] The case presented in this article showed native bone of 7-8 mm[20] at both the sites, as a result of which implant placement could be satisfactorily done even without placement of bone graft.[19]

### Case VI:

**Figure 6:**
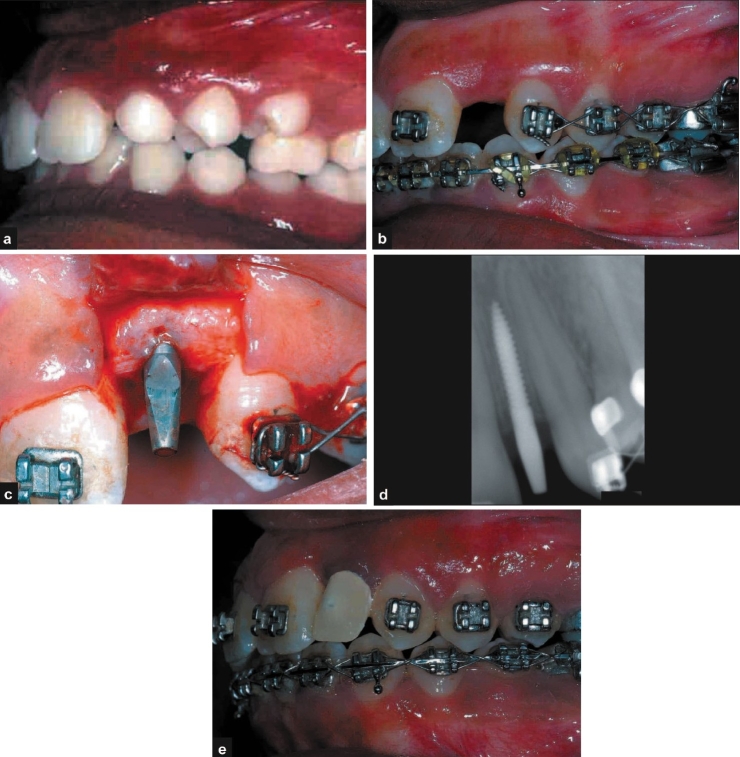
a, b) Pre-operative photographs; c, d) operative photograph and radiograph; e) post-operative photograph

An 18-year-old female patient was referred from the Department of Orthodontics for restoration with implant in relation the tooth number 22, the space for which was gained orthodontically [Figure 6a and b]. Clinical and radiological evaluation was performed and it was decided to perform a single stage implant placement$ (2.4 × 10 mm) [Figure 6c and d]. The patient received temporary acrylic crowns on the same day. The patient was recalled for prophylaxis after two and four weeks and at third month. The clinical and radiographic appearances at three months show good aesthetic result and acceptable osseo-integration of the implants [Figure 6e].

## DISCUSSION

Orthodontic tooth movement is a physiological method of regenerating bone in inadequate sites without the use of any bone replacement grafts.[21] With an ever increasing number of patients seeking both adult orthodontics and implant rehabilitation, the role of an orthodontist in treatment planning for implant placement has become important.

## CONCLUSION

With the ever increasing popularity of implant supported prosthesis and increasing patient demand for good esthetics, the need for greater use of the single stage implants has become tremendous. It is, therefore, necessary for the clinician to be able to clearly distinguish areas were these implants may be successfully restored without any undesirable post-operative sequelae. The cases presented in this paper were all restored successfully with good patient acceptance.

## Footnotes

* Regen Biocement and Hydrase

# Periocol GTR Membrane

$ TRX-OP Single-Piece Implant (LifeCare)

$$ TRX-BA Single-Piece Overdenture Implant (LifeCare)

## References

[1] Hoffmann O, Beaumont C, Zafiropoulos GG (2006). Immediate Implant Placement: A Case Series. J Oral Implantol.

[2] Weischer T, Kandt M, Reidick T (2005). Immediate loading of mandibular implants in compromised patients: preliminary results. Int J Periodontics Restorative Dent.

[3] Schwartz-Arad D, Laviv A, Levin L (2007). Survival of immediately provisionalized dental implants placed immediately into fresh extraction sockets. J Periodontol.

[4] Juodzbalys G, Wang HL (2007). Soft and hard tissue assessment of immediate implant placement: A case series. Clin Oral Implants Res.

[5] Donati M, La Scala V, Billi M, Di Dino B, Torrisi P, Berglundh T (2008). Immediate functional loading of implants in single tooth replacement: a prospective clinical multicenter study. Clin Oral Implants Res.

[6] Fritz ME, Jeffcoat MK, Reddy M, Koth D, Braswell LD, Malmquist J (2001). Implants in regenerated bone in a primate model. J Periodontol.

[7] Ioannidou E, Doufexi A (2005). Does Loading Time Affect Implant Survival? A Meta-Analysis of, 1,266 Implants. J Periodontol.

[8] Aral A, Yalçin S, Karabuda ZC, Anil A, Jansen JA, Mutlu Z (2008). Injectable calcium phosphate cement as a graft material for maxillary sinus augmentation: An experimental pilot study. Clin Oral Implants Res.

[9] Harvey BV (2007). Optimizing the Esthetic Potential of Implant Restorations Through the Use of Immediate Implants With Immediate Provisionals. J Periodontol.

[10] Zeren KJ (2006). Minimally invasive extraction and immediate implant placement: the preservation of esthetics. Int J Periodontics Restorative Dent.

[11] Jalbout ZN, Tarnow DP (2001). The implant periapical lesion: four case reports and review of the literature. Pract Proced Aesthet Dent.

[12] Tözüm TF, Sençimen M, Ortakoğlu K, Ozdemir A, Aydin OC, Keleş M (2006). Diagnosis and treatment of a large periapical implant lesion associated with adjacent natural tooth: A case report. Oral Surg Oral Med Oral Pathol Oral Radiol Endod.

[13] Covani U, Cornelini R, Barone A (2007). Vertical crestal bone changes around implants placed into fresh extraction sockets. J Periodontol.

[14] Marcus SE, Drury TF, Brown LJ, Zion GR (1996). Tooth retention and tooth loss in the permanent dentition of adults: United States, 1988-1991. J Dent Res.

[15] Mecall RA, Rosenfeld AL (1991). Influence of residual ridge resorption pat-terns on implant fixture placement and tooth position. 1. Int J Periodontics Restorative Dent.

[16] Eckert SE, Carr AB (2004). Implant-retained maxillary overdentures. Dent Clin North Am.

[17] MacEntee MI, Walton JN, Glick N (2005). A clinical trial of patient satisfaction and prosthodontic needs with ball and bar attachments for implant-retained complete overdentures: three-year results. J Prosthet Dent.

[18] Wallace SS, Froum SJ (2003). Effect of maxillary sinus augmentation on the survival of endosseous dental implants. A systematic review. Ann Periodontol.

[19] Sohn DS, Lee JS, Ahn MR, Shin HI (2008). New bone formation in the maxillary sinus without bone grafts. Implant Dent.

[20] Lundgren S, Andersson S, Gualini F, Sennerby L (2004). Bone reformation with sinus membrane elevation: A new surgical technique for maxillary sinus floor augmentation. Clin Implant Dent Relat Res.

[21] Biggs J, Beagle JR (2004). Pre-implant orthodontics: achieving vertical bone height without osseous grafts. J Indiana Dent Assoc.

